# Editorial. Life Sciences in an Integrated Curriculum: Starting the Conversation

**DOI:** 10.15694/mep.2017.000063

**Published:** 2017-04-03

**Authors:** Barbara A. Jennings, Iain Keenan

**Affiliations:** 1 Norwich Medical School; 2 School of Medical Education

**Keywords:** Life Sciences, Integrated Curriculum, Learning Gain, Anatomy, Genetics

## Abstract

This article was migrated. The article was marked as recommended.

Editorial, no abstract required.

## Aims

More than 200 articles have been published online in the new MedEdPublish journal, but we found that surprisingly few describe the learning and teaching of Life Sciences ( 1- 7). This observation could reflect the published research within the wider medical education community. In order to enhance and expand upon the current literature in this area, the editors invite your contributions to this themed-issue. We welcome commentaries, opinions, case studies, reviews and full research papers.

While there may be adequate Life Sciences teaching sessions within undergraduate medical curricula, it is important to address the nature, quality, timing and integration of their delivery. There are many discipline-specific curricula outlined within the published literature that focus on competencies and learning outcomes ( 8- 12), but although biological sciences are a cornerstone of training, limited attention has been given to the development of evidence-based approaches for the teaching of these core sciences. Even for anatomy, a discipline that is well-represented within the educational literature, concerns over the methodological rigour of the vast majority of studies have been raised after systematic review ( 13). Furthermore, only a limited repertoire of the wide variety of available learning and teaching methods have been investigated in well-designed studies.

**Figure 1.**
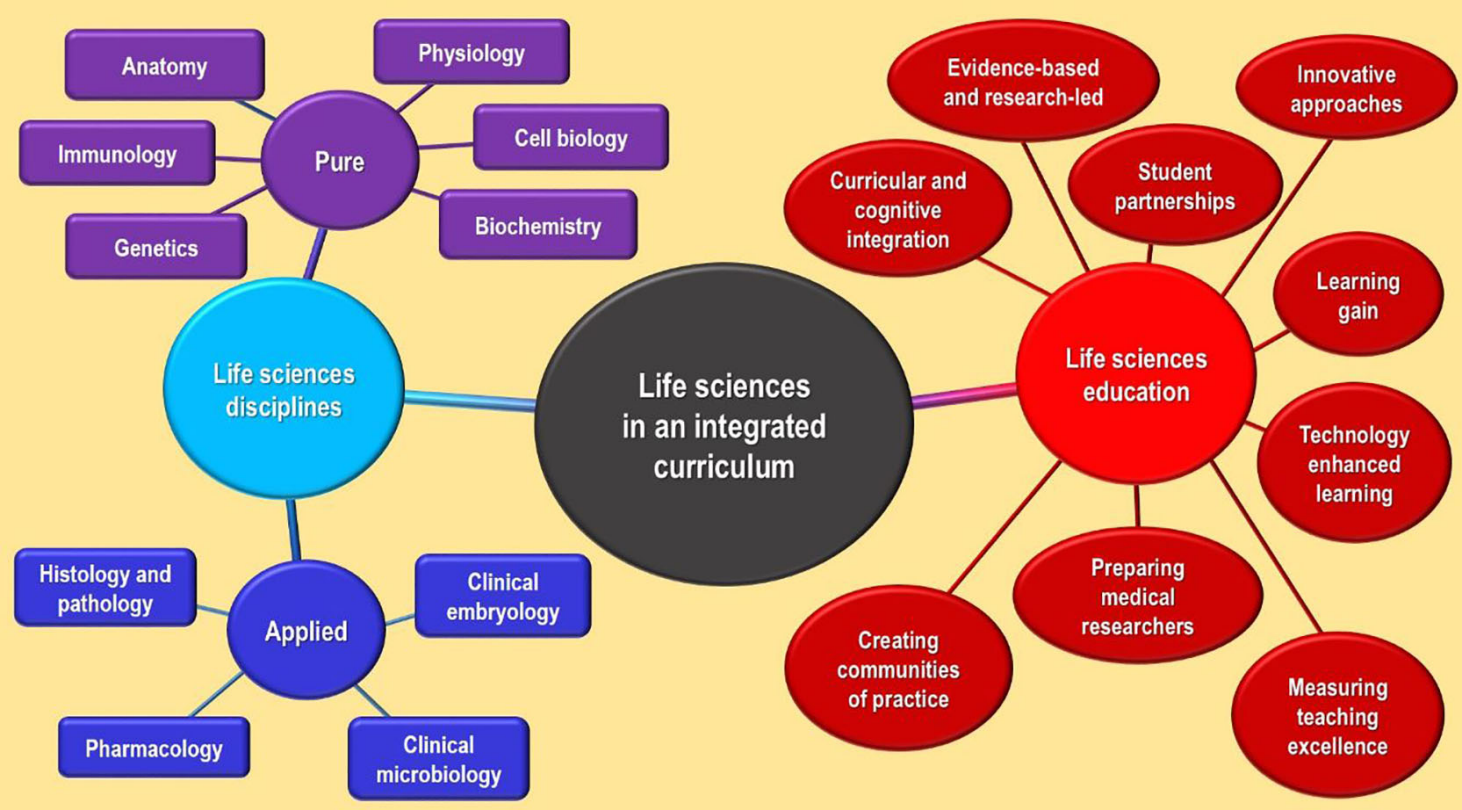
An illustration of the Life Sciences and educational topics for this themed-issue of MedEdPublish

In this themed-issue, we encourage the submission of articles from basic science teachers and medical educators that describe innovative and effective ways to support medical students in their study of the Life Sciences. The scope of this edition includes disciplines such as anatomy; genetics and physiology; in addition to the more applied fields, such as clinical microbiology; pharmacology; and histology (for a more comprehensive list please see the illustration in Figure 1).

We propose an exploration of the following topics within Life Science education:

## (1) Learning Gain, Innovation and Teaching Excellence

Life Science teaching may be more strongly associated with the didactic lecture than other strands of clinical education, where the principles of social learning have been embraced. However, in 2009 the Harvard Physicist Eric Mazur provided a reasoned argument for saying farewell to the traditional lecture as a mode of delivery for his teaching ( 14). He had come to his conclusions reluctantly because he enjoyed delivering his lectures and student evaluations had always been positive. However, systematic testing of *the lecture* against new strategies, showed that learning-gain in his students nearly tripled by using a student-centred approach coupled with interactive learning. Mazur noted that this is not only of value for the gain of conceptual knowledge but also for improving those problem-solving skills that we know are so important for clinical reasoning and diagnostic accuracy. A recent meta-analysis of 225 primary studies supports Mazur’s conclusions that active learning methods can increase assessment performance and should be utilised as the standard control for educational experimental evaluations ( 15).

Last year AMEE president Trudie Roberts, described some of the challenges of defining excellence in medical education ( 16). We can focus on student evaluations of teaching events, teacher development, and their technical competence, but overall strategies for measuring effectiveness should also consider the impact on our students’ learning. This issue was addressed by a *Nature* features editor, M. Mitchell Waldrop, in his review of studies about the teaching of university science ( 17) and conclusions were very damning for the didactic lecture, but encouraging of problem-oriented learning and innovative flipped-lecture approaches: *“At this point it is unethical to teach in any other way”.*

## (2) Research-Led Teaching and Evidence-Based Practice

It is important that a research-led approach is utilised and good evidence is sought to support the wide inclusion of learning methods in Life Sciences, as opposed to reliance on trends, assumptions and accepted truths; where the inherent validity of methods under investigation is taken for granted. Systematic review can be used to evaluate existing studies of educational interventions, but conclusions depend on the quality of primary studies. For example, there is a large amount of literature advocating the use of ultrasound in undergraduate anatomy teaching, based on specific rationales. However, a critical appraisal of the evidence suggests that there is minimal support to justify this ( 18). The authors of this systematic review found that there was weak or inconsistent evidence to support the premise that ultrasound could improve outcomes such as anatomy learning; physical examination and diagnostic skills; or patient care. In some of the primary studies identified, self-reported student perceptions alone were utilised as the basis of such claims. Because of the prevalence of these established rationales within the literature, it is proposed that the use of ultrasound may have been legitimised without sufficient evidence. The authors justifiably raise the concern that such situations could encourage further use of particular learning methods that have limited evidence to support their value, while discouraging further research.

## (3) Integration and Creating a Community of Practice

Integration of the Life Sciences into medical curricula is essential so that effective links can be made between an understanding of biology and clinical practice. But how confident are we that our strategies for integrating basic and clinical sciences are leading to optimal learning-gain for our students? A critical narrative review has shown that studies investigating this integration are often inadequately designed, and has highlighted the importance of considering the cognitive aspects of student learning when integrating basic and clinical sciences. The authors therefore suggest it is unhelpful to rely too heavily on terms such as horizontal, vertical and longitudinal integration and propose that cognitive integration should instead by emphasised. They have subsequently suggested that emphasising conceptual knowledge within a variety of relevant contexts can enhance the integration of basic science knowledge into clinical reasoning ( 19).

Creating a holistic curriculum with effective integration of life, population, and social sciences with clinical practice requires collaboration and creativity within and between institutions. Integration of curricular strands also relies on relationships between faculty members who will have very different priorities and perspectives. At the AMEE 2016 conference in Barcelona, Martin Fischer shared the challenges of integrating teaching between different professional groups at the institutional level and offered ideas for overcoming barriers that exist between the faculty members who deliver basic and clinical sciences ( 20). A prevailing open-science and open-education culture also facilitates collaboration. This is illustrated by the sharing of big-data sets in meta-analyses, the growth of the massive-open-online-course (MOOC) and tools for technology-enhanced-learning (TEL). TEL includes the use of social media for professional networking, dissemination of good practice and as a learning and teaching approach ( 21). The development of one online community of practice for teachers was also presented at AMEE 2016 by Diana Laurillard when she described the Learning Design Support Environment ( 22). This platform allows teachers to design new teaching sessions and, with the help of feedback from the software, to visualise the type and quality of the experiences their learners will have. Adapting the design after reflection is straightforward, and so is the creation and development of learning designs with other teachers. The editors hope that this themed-issue of MedEdPublish could inspire the sharing of innovative lesson plans, modules and tools for TEL.

## (4) Preparing Medical Researchers and Student Partnerships

To improve healthcare, we rely on medical graduates to contribute to innovations that result from medical research. Concerns have been raised about the erosion of curricular time devoted to the development of laboratory and research skills within the modern medical curriculum ( 23) and its potential impact on training the next generation of clinical academics.

Some fields of research are evolving very rapidly, such as genetic medicine. Teachers see the challenge that learners face as they navigate their way through the relevant literature and data that is accumulating exponentially. However, evaluation of translational genomics from discovery phase to health impacts, reveals that most human genomics research may be lost in translation; with studies of genetic healthcare applications comprising less than one percent of all published literature ( 24). This suggests two roles for the teacher of genetics (and other sciences); first to equip learners with the necessary critical appraisal skills, so that they can evaluate interventions that appear to demonstrate clinical utility throughout their careers. But also to adequately prepare undergraduates to contribute to the timely application of new tests and treatments with proven utility. Utilising approaches for the development of undergraduate research and transferable skills through student partnership is also recommended ( 25).

## Starting the Conversation

We aim to encourage contributions to this themed-issue of the journal from teachers and their undergraduate students, in addition to curriculum planners; postgraduate deans; and representatives of professional bodies who oversee postgraduate medical training.

In his first editorial of 2016, Richard Hays explained several aspects of publishing innovation offered by MedEdPublish ( 26). The area we particularly want to highlight to the authors and readers of this edition, is the facility to create a rapid-response discussion thread around each article. This editorial has been produced with the intention of starting a conversation regarding the delivery of an integrated and evidence-based Life Sciences curriculum for medical education.

## Take Home Messages

Editorial, not applicable.

## Notes On Contributors

Dr Barbara Jennings joined Norwich Medical School as part of the inaugural team when it was established in 2002, and is a Senior Lecturer in the Medical Education department. She is the academic lead for the genetics curriculum, and for faculty continuous-professional-development. Barbara has a background in cancer research and in clinical molecular diagnostics. Her research spans cancer genetics, genetic epidemiology and pharmacogenetics. Barbara is a member of the MedEdPublish editorial board and is Senior Fellow of the Higher Education Academy. Please see Barbara’s twitter account; @GeneticsMBBS

Dr Iain Keenan is a Lecturer in Anatomy within the School of Medical Education at Newcastle University. Iain has a research background in Life Sciences and is currently an academic lead for anatomy teaching and curriculum officer for the undergraduate medical degree programme at Newcastle. Iain also contributes to postgraduate medical education, medical sciences, physician associates and clinical training programmes. Iain is an active investigator in medical education with a particular interest in innovative and creative learning methods in anatomy education and is councillor, Website, Media and Communications Officer and Social Media Editor for the Anatomical Society. Iain is a fellow of the Higher Education Academy. Please see Iain’s twitter account; @dr_keenan

## Declarations

Barbara Jennings and Iain Keenan are guest Theme Editors for the AMEE MedEdPublish themed issue, Life Sciences in an Integrated Curriculum.
