# Supplementary material for: Editorial. Life Sciences in an Integrated Curriculum: Starting the Conversation
Source: MedEdPublish (2016). 2017 Apr 3;6:63. [Version 1] doi: 10.15694/mep.2017.000063 (PMC10885250; doi:10.15694/mep.2017.000063)
Supplement: Peer review history

# Reviewer report: Reviewer response for version 1

**DOI:** 10.21956/mep.19280.r28045

**Published:** 2017-04-07

**Authors:** Hilary Burton

**Affiliations:** 1 PHG Foundation

This review has been migrated. The reviewer awarded 5 stars out of 5 Thanks for this thoughtful article and for highlighting the importance of good learning in Life Sciences as preparation for medical practice. For the last few years I have been leading a UK Royal College of Physicians group on developing genomics in mainstream practice and there is a recurring theme that today's practitioners are ill prepared for 21st century personalised medicine. As your article highlights, the science is moving quickly and new doctors must be equipped with the basic knowledge, but more importantly with the skills to keep up to date and connected with new developments. All of them will be using genomics in their future diagnoses and clinical decision making. I would love to hear more about your experience in progressing understanding of genomics from fact-based to the development of skills that prepare students to be adaptive and able to apply knowledge critically within their practice.

Reviewer Expertise:

NA

No decision status is available

# Reviewer report: Reviewer response for version 1

**DOI:** 10.21956/mep.19280.r28044

**Published:** 2017-04-07

**Authors:** Mohamed Al-Eraky

**Affiliations:** 1 University of Dammam

This review has been migrated. The reviewer awarded 4 stars out of 5 I concur with the authors to expand the scope of the conversation to include stakeholders from professional bodies, beyond medical schools. They represent the potential employers and accreditors of medical graduates. Their views are more comprehensive to address, not only technical competencies, but also the interpersonal skills and attitudes of graduates. That said, in the past decade scholarly articles indicated a rich number of activities where behaviors of professionalism can be taught, practiced, and rewarded in the context of basic sciences. Till recently, it was assumed that medical professionalism can be taught only in clinical contexts. We invite science teachers to discuss more opportunities to tackle complex outcomes (e.g. medical professionalism and ethics) in content-based courses like: anatomy.

Reviewer Expertise:

NA

No decision status is available

# Reviewer report: Reviewer response for version 1

**DOI:** 10.21956/mep.19280.r28047

**Published:** 2017-04-04

**Authors:** Richard Hays

**Affiliations:** 1 James Cook University

This review has been migrated. The reviewer awarded 5 stars out of 5 Thanks to the authors for starting the conversation about an important and contemporary topic. Science is essential to medical practice, and yet how we deliver that scientific platform underpinning quality care remains controversial, with wide variations in curriculum design. A couple of additional and really important issues are: 1. The nature of 'integration' and 2. assessment in an integrated curriculum. 'Pseudo-integration' and poor assessment may lead to either a very heavy 'hidden' curriculum or very shallow learning that may not prepare graduates adequately for future scientific progress. Further, curriculum time has been eroded in many areas, not just life sciences. Pathology, research experience and even clinical placements have also been trimmed in the rather elusive drive for efficiency gains as potential curriculum content expands rapidly. This may not always be bad, as sometimes teaching less allows more to be learned, but achieving agreed graduate outcomes should not be an accidental process. Please join the conversation!

Reviewer Expertise:

NA

No decision status is available

# Reviewer report: Reviewer response for version 1

**DOI:** 10.21956/mep.19280.r28043

**Published:** 2017-04-03

**Authors:** Trevor Gibbs

**Affiliations:** 1 AMEE

This review has been migrated. The reviewer awarded 5 stars out of 5 This is the start of a very important theme, one that needs addressing and one that must be discussed in an appropriate manner- I fully support the approach taken by the authors and the four broad approaches.Having seem many curricula in action around the world, I have seen, probably like the authors, many attempts to integrate the life sciences, or as known to many the basic sciences, into curricula, only to fail because of the out-dated methods of assessment used. I wonder therefore if anyone has tackled the issue from the other side and started with a more integrated, real-world, assessment package, doing away with MCQs altogether and introducing a much more applied sciences approach.I look forward to reading the papers in this theme and hopefully read some innovative ways of integrating the life sciences

Reviewer Expertise:

NA

No decision status is available

# Reviewer report: Reviewer response for version 1

**DOI:** 10.21956/mep.19280.r28046

**Published:** 2017-04-03

**Authors:** John Cookson

**Affiliations:** 1 University of Worcester

This review has been migrated. The reviewer awarded 5 stars out of 5 I fully support the aims of this editorial and the pursuit of a more integrated curriculum across the life sciences and the social and population sciences and clinical experience. For me, a key concept was understanding the role of clinical experience in the early years. This is usually seen as acquiring real ‘doctoring skills’ and so it should be. At least as important, however, is the stimulus it gives to basic science learning if managed appropriately. If, for example in a PBL course, students meet the same kind of patient ‘for real’ that they have been studying in the classroom at the end of each week then ‘all the lights go on’. So the usual concept of learning the basic science before going on to apply it is turned around. The patient sets the agenda for the learning. I’ve heard it suggested that a 5-year medical course should consist of 3 years clinical followed by 2 years of basic science. After 3 years of seeing patients you know what you really need to know. Perhaps that’s too radical but it reflects what doctors actually do when faced with a difficult problem which they can’t solve by pattern recognition; they go back to the basic science.

Reviewer Expertise:

NA

No decision status is available
